# Identification of unknown acid-resistant genes of oral microbiotas in patients with dental caries using metagenomics analysis

**DOI:** 10.1186/s13568-021-01199-4

**Published:** 2021-03-06

**Authors:** Xi Cheng, Fuming He, Ping Sun, Qianming Chen

**Affiliations:** The Affiliated Hospital of Stomatology, School of Stomatology, Zhejiang University School of Medicine, and Key Laboratory of Oral Biomedical Research of Zhejiang Province, Hangzhou, 310006 Zhejiang China

**Keywords:** Microbiome, Acid resistance, Metagenomic, Dental caries, Oral probiotics

## Abstract

Acid resistance is critical for the survival of bacteria in the dental caries oral micro-environment. However, there are few acid-resistant genes of microbiomes obtained through traditional molecular biology experimental techniques. This study aims to try macrogenomics technologies to efficiently identify acid-resistant genes in oral microbes of patients with dental caries. Total DNA was extracted from oral microbiota obtained from thirty dental caries patients and subjected to high-throughput sequencing. This data was used to build a metagenomic library, which was compared to the sequences of two S*treptococcus mutant* known acid-resistant genes, *danK* and *uvrA*, using a BLAST search. A total of 19 and 35 unknown gene sequences showed similarities with *S. mutans uvrA* and *dnaK* in the metagenomic library, respectively. Two unknown genes, *mo-dnaK* and *mo-uvrA*, were selected for primer design and bioinformatic analysis based on their sequences. Bioinformatics analysis predicted them encoding of a human heat-shock protein (HSP) 70 and an ATP-dependent DNA repair enzyme, respectively, closely related with the acid resistance mechanism. After cloning, these genes were transferred into competent *Escherichia coli* for acid resistance experiments. *E. coli* transformed with both genes demonstrated acid resistance, while the survival rate of *E. coli* transformed with *mo-uvrA* was significantly higher in an acidic environment (pH = 3). Through this experiment we found that identify unknown acid-resistant genes in oral microbes of patients with caries by establishing a metagenomic library is very efficient. Our results provide an insight into the mechanisms and pathogenesis of dental caries for their treatment without affecting oral probiotics.

**Supplementary Information:**

The online version contains supplementary material available at 10.1186/s13568-021-01199-4.

## Introduction

Dental caries, one of the most common oral diseases, is not only capable of destroying the hard tissues of teeth, but also acts as an initial stimulus for multiple systemic diseases. Accordingly, many studies have focused on the prevention and treatment of dental caries. Microbial colonization in the oral cavity is known to be critical to the pathogenesis of caries (Rosier et al. 2018). Due to the complex biofunctions and compositional diversity of oral microbiota, the specific causes of dental caries have yet to be fully elucidated. In the oral micro-environment of patients, specific functions are required for the survival and reproduction of pathogenic bacteria (e.g. environmental acidity, microbial acid resistance, and adhesion properties) (Peterson et al. 2014). Among these, acid resistance has proven to be critical, particularly within the first 20 min after food intake, when the pH of the oral micro-environment drops to pH 3 from pH 7 (Dodds et al. 1986), providing a harsh environment for the survival of pathogenic bacteria. Thus, from the perspective of personalized medicine and clinical applications, acid resistance in oral microbiota must be taken into account for the study of dental caries.

At present, due to the limitations of traditional genetic and molecular biotechnologies (based on the in vitro culture of bacteria), few acid resistance genes in oral microbiota have been identified and studied. In the 1980s, Loesche et al. reported *Streptococcus mutans* as the main pathogen responsible for caries, which was found in the oral cavity of almost all caries patients (Loesche et al. 1986). Subsequently, extensive research was considered on the acid-producing and acid-resistant properties of *Streptococcus mutans*, which resulted in the identification of several acid-resistant genes, including *ffh*, *uvrA*, and *dnaK* (Jin et al. 2011). These achievements can be attributed to the effective breeding of *S. mutans* in laboratories. However, 60% of oral microbiotas cannot be cultured in vitro. Based on 16sRNA rRNA high-throughput sequencing, scholars found that non-cultivable oral microbiotas are widely involved in the formation of caries, including *Selenomonas* and *Neisseria* (Wade et al. 2013). Meanwhile, except for *Streptococcus mutans*, we have limited knowledge of the acid resistance and regulatory genes of caries pathogens, especially in terms of non-cultivable oral microbiotas. This forms the basis for our experiments, with the aim to identify unknown acid-resistant genes using metagenomics analysis.

Microbial metagenomics is a recent and popular molecular method that is able to recognize all of the currently known microbial genes and target functions involved. In addition, it can be used to identify the genetic information of unknown microbiotas (Gilbert et al. 2010). Microbial metagenomics has been extensively used in various fields in studies, including studies on the composition of natural environmental microbiomes (Agarwal et al. 2017) and for the extraction of pharmaceutical enzymes from bear feces microbiomes (Song et al. 2017). However, few studies have used this method to study the microbiota of dental caries. In this study, we used metagenomics to identify unknown acid resistance genes in caries patients to provide a new point of entry for the study of the pathogenesis of caries and their treatment. We constructed a local metagene library by extracting the total DNA from the oral microbiota of dental caries patients. BLAST (Basic Local Alignment Search Tool) was used to identify unknown genes with sequences similar to the sequences of known *Streptococcus mutans* acid-resistant gene. Using bioinformatics analysis, we predicted two positive clones (*mo-dnaK*, *mo-uvrA*) to encode heat-shock protein (HSP) 70 and ATP-dependent DNA repair enzyme.

## Materials and methods

### Ethics statement

Informed consent was obtained from the patients, as well as from family members who were willing to provide experimental assistance. The Ethics Committee of the People’s Hospital of Leshan approved the design, agreements, and informed consent of our study (Leshan City, Sichuan Province, China). Ethics number: 20180112009.

### Sample collection

Saliva samples were collected from thirty caries patients aged 55–60 years who visited the Department of Stomatology at the People’s Hospital of Leshan in October 2018. (Collected when the author was an employee of this hospital). The inclusion criteria used were obtained from Zhang. etc. (Zhang et al. 2018). Patients included those who (1) had no systemic diseases, (2) had not used any antibiotic within the last three months, (3) had not used fluoride locally and had no history of oral filling, (4) with tooth decay scores of > 5, and (5) had more than 24 remaining teeth. Patients were not allowed to eat, drink, or clean their teeth before sampling (within 2 h) (Zhang et al. 2018). At the time of sampling, 2 ml of saliva per patient spit in a soft test tube. After sampling, all saliva is placed in a sterile beaker and mixed well. Next, it was equally divided into three portions. Samples were stored in dry ice (− 80 °C) and sent to the laboratory for testing.

### DNA extraction, library construction, and metagenomic sequencing

The microbiological DNA was extracted from the oral microbial samples using an E.Z.N.A.® DNA Kit (Omega Bio-Tek, Norcross, GA, United States), according to the manufacturer’s instructions. The concentrations and purity of extracted DNA was determined using a TBS-380 Mini-Fluorometer and NanoDrop2000, respectively. The quality of the extracted DNA was assessed on a 1% agarose gel. DNA extracts were fragmented to an average size of approximately 300 bp using Covaris M220 (Gene Company, Shenzhen, China) for the paired-end library, which was constructed using the TruSeqTM DNA Sample Prep Kit (Illumina, San Diego, United States). Adapters containing the full complement of sequencing primer hybridization sites were ligated to the blunt-end fragments. Paired-end sequencing was performed on an Illumina HiSeq4000 platform (Illumina, San Diego, United States) at Majorbio Bio-Pharm Technology Co., Ltd. (Shanghai, China) using a HiSeq 3000/4000 PE Cluster kit and HiSeq 3000/4000SBS kit, according to the manufacturer’s instructions (http://www.illumina.com). Sequence data associated with this project have been deposited in the NCBI Short Read Archive database (NCBI accession no. PRJNA533520).

### Sequence quality control and genome assembly

First,Sickle (https://github.com/najoshi/sickle) was used to remove low quality reads (length < 50 bp,quality < 20,or multiple bases). Then,SeqPrep (https://github.com/jstjohn/SeqPrep) was used to strip the adaptor sequence from the 3′and 5′ ends of the end reads. These reads were compared to the human genome (version 38)using the BWA tool (http://bio-bwa.sourceforge.net). Any matches related to thereadand the paired reads were deleted. MEGAHIT tool (https://github.com/voutcn/megahit) was used to assemble the metagenomic data. This method uses aconcisede Bruijn diagram. Convolutions longer than 300 bp were selected as the final asembly result and then used for further gene prediction and annotation.

### Selection of acid-resistance clones and bioinformatics analysis

**Fig. 1:**
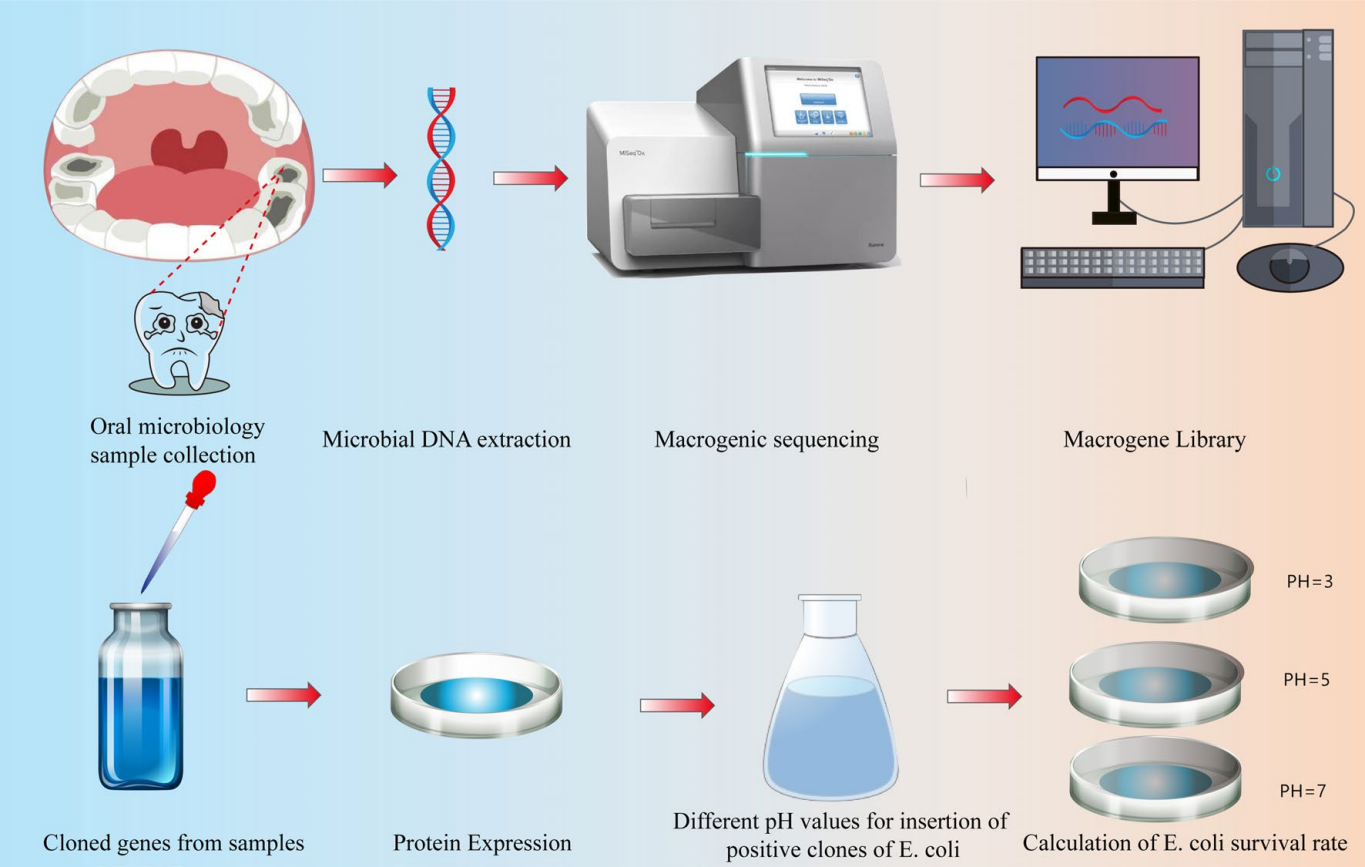
Experimental Mind Maps

The mutants of the *Streptococcus* acid-resistance genes *dnaK* and *uvrA* were identified using the NCBI database (Fig. 1a). Using DNAman v6.0 and Vector NTI Advance 11.5.1 software, a potential open reading frame (orf) with a start codon and three stop codons was predicted and analyzed (start codon: ATG; stop codons: TGA, TAA,TAG). Additionally, the online NCBI server (https://blast.ncbi.nlm.nih.gov/Blast.cgi) and Blast N and X (essentiallocal alignment search tool) were used to align the nucleotide sequences and transcribe theamino acid sequences. In turn, the conserved domains (CD) search module (https://www.ncbi.nlm.nih.gov/Structure/cdd/wrpsb.cgi) was used to analyze the conserved domains.As a result, the predicted ORFs were obtained and analyzed, similar to knownor unknown proteins of over 100 bp in length. The Protein families (http://xfam.org/) and Blocks (http://www.ebi.ac.uk/interpro/) databases were used to compare the results of the BLAST searches.

### Cloning and expression of mo-urvA and mo-dnaK genes

**Fig. 2:**
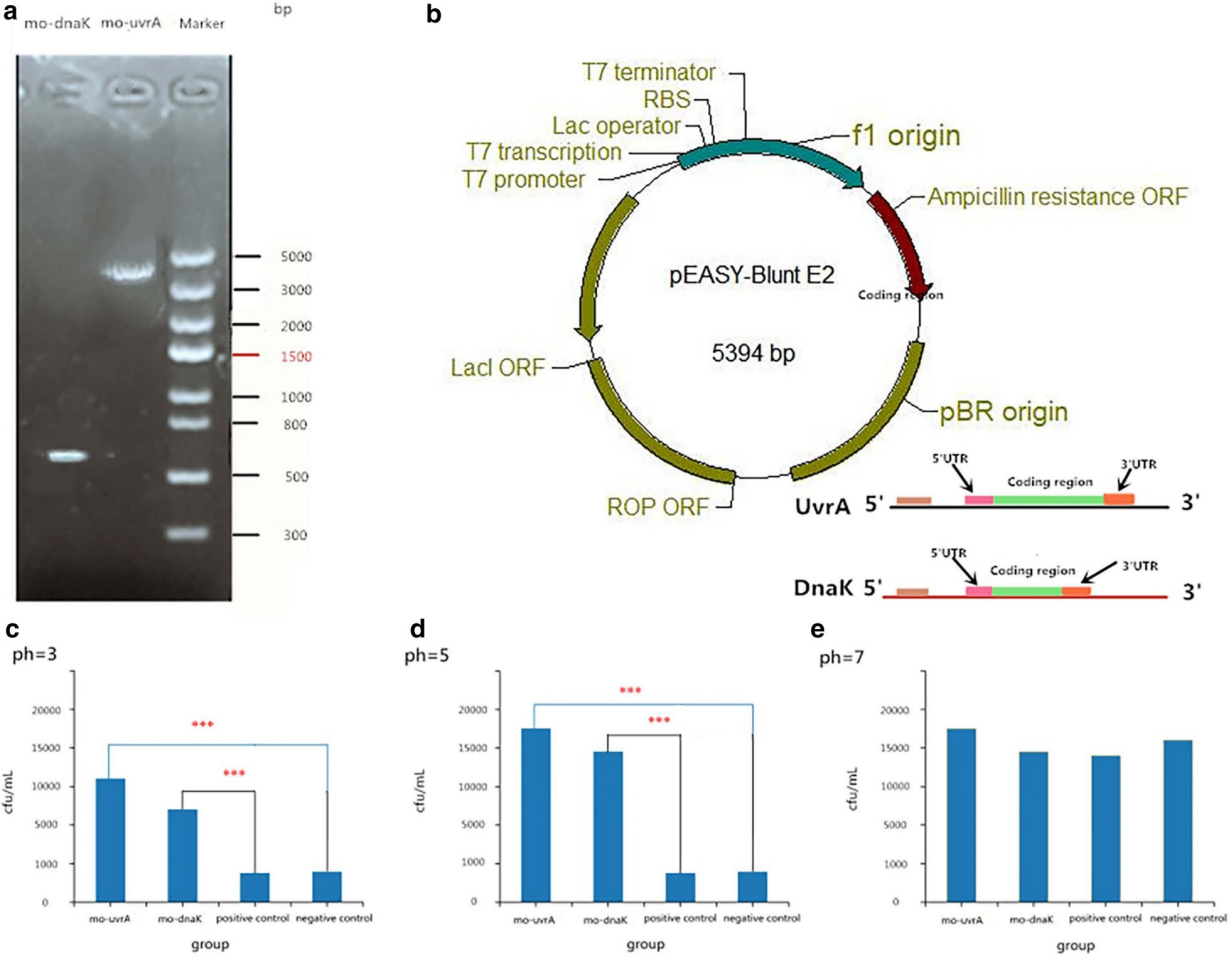
This picture has five part. **a** The gel electrophoresis pattern after PCR amplification of the two genes. **b** The structure of the plasmid used in the experiment. **c**, **d**, **e** Acid resistance analysis results, CFU index at three different pH values

Based on the BLAST results, the full sequence of the genes for *mo-urvA1* and *mo-dnaK1* were harvested (Fig. 1b). Multiple sequence comparisons and homology analyses were conducted by searching the GenBank using DNAMAN software. The cDNA sequences of mo-urvA and mo-dnaK were used to design the primers for the two genes: for *mo-urvA1*, forward primer 5′-ATGCAAGATAAGTTAGTGATTC-3′, and reverse primer 5′-TTATGTCAATTTTTCTTTCAAATATTGTCC-3′; for *mo-dnaK1*, forward primer 5′-ATGGCTGTCGGGCGACCG-3′ and reverse primer 5′-TAAGTCGCCATGTTCCTTCAGCGAT-3′,using high-fide litypoly merase TranStart Fast PfuFly. The size of these two genes was found to measure up to 500–3,000 bp, according to DNA polymerase PCR amplification (Fig. 2a). By recycling the target fragments, *mo-urvA* and *mo-dnaK* were built into the carrier pEASY-Blunt E2, expressed, and transformed into E. coli (Competent E. coli) Trans1-T1 cells (Fig. 2b). The positive clones for PCR detection were sequenced, and the plasmid was harvested from clones positive for the correct sequences. After harvesting the correct plasmid, the target fragments of *mo-urvA* and *mo-dnaK* were recovered and purified before being ligated into the prokaryotic expression vector pEASY-BluntE2 (Fig. 2b) and transferred into E. coli Trans1-T1 using a heat-shock method. The plasmids exhibiting the correct sequences were used in subsequent experiments with the corresponding strains.

### Acid-resistance assay and statistical analysis

The resulting competent E. coli cells were split into 4 groups: negative control, positive control, *mo-urvA* cloned, and *mo-dnaK* cloned groups. Isopropyl β-D-1-thiogalactopyranoside (IPTG) was added to the control, *mo-urvA* cloned, and *mo-dnaK1* cloned groups. Both the positive clone E. coli group and the negative/positive E. coli control group were activated and inoculated for 1 h in 5 mL LB-Amp broth at 37ºC. Lysogeny broth (LB) at various pH values (pH 3, 5, and 7) was produced. Then, 25 mL of the medium at each pH value was added to separate bottles. The bacterial culture was then transferred into the bottles with LB medium at the different pH values and incubated at 37ºC for 12 h. After incubation, 1 mL samples of the liquid medium were added to solid medium. The survival percentage was calculated by dividing the final number of CFU/mL, as illustrated in Fig. 2c, d, and e. The data were analyzed with SPSS 19.0 and the level of significance were analyzed by one way analysis of variance (ANOVA). *P* < 0.05 were considered statistically significant.

## Results

### Subjects and samples

Saliva samples collection from 30 patients' oral cavity were mixed and then divided into three parts. There were placed in big tube and sequenced. The final three samples weighed 30.2, 31.1, and 32.8 mg, (Named P1, P2, P3 respectively).

### Microbiome taxonomic composition

**Fig. 3:**
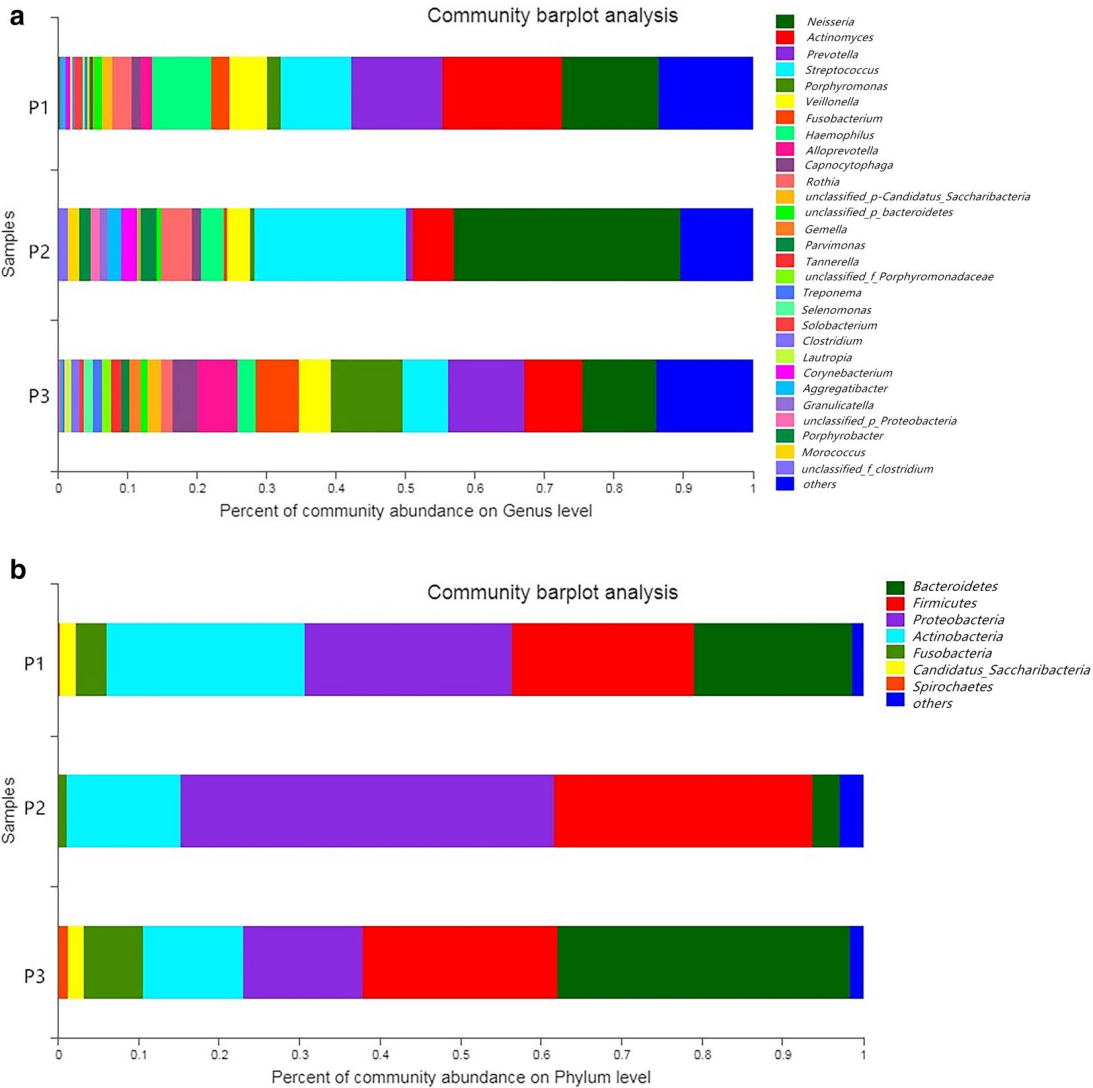
The three oral samples microbiomes community composition analysis on phylum level and genus level

The reads were compared with the host genome sequences using the corresponding software. Any reads displaying high similarity were removed. The pollution-free texts applied for the subsequent analysis were 34,421,160, 13,118,829, and 60,148,375 (Table 1). As observed in the community composition analysis (Fig. 3b), the oral samples obtained from dental caries patients were primarily composed by members of the following phyla: *Bacteroidetes, Firmicutes*, and *Proteobacteria*. Moreover, at the genus level (Fig. 3a), *Neisseria, Actinomyces, Prevotella*, and *Streptococcus* were found to be the most abundant phyla in the samples.

**Table 1:** Metagenomic sequencing data

Part	Optimized reads	Optimized bases (bp)	Percent in raw reads (%)n	Percent in raw bases (%)
P1	13,118,829	1,959,777,890	14.72	14.56
P2	34,421,160	5,167,931,370	34.45	34.26
P3	60,148,375	9,033,787,469	59.06	58.75

### Basic local alignment search tool search result

In the oral metagenomics library for dental caries, a total of 4,246,651 sequences were obtained (Table 1). A total of 35 unknown gene sequences with structures similar to those of *dnaK* were found (Fig. 1c). P3_k97_439164_gene_3, with a length of 1956 base pairs, a score of 418 bits (226), and 81% similarity to the original *danK* gene (denoted as *mo-danK*) was chosen for further experiments (Fig. 1b). For the *uvrA* gene, 19 unknown gene sequences with structures similar to the original gene were found using local BLAST analysis (Fig. 1c). The gene P1_k97_2690568_gene_1 (Fig. 1b), with a length of 2826 base pair, a score of 4089 bits (2214), and 90% similarity to the original *uvrA* (denoted as *mo-uvrA*) was used for subsequent analyses (Fig. 1b).

### Bioinformatics analysis of acid-resistant clones

The sequences of the two unknown genes are shown in Fig. 1b. The results of the bioinformatics analysis demonstrated the genetic similarity, potential open reading frames, phylogenetic relationships, and functional presumptions, as summarized in Table 2. As shown in Table 1, the two unknown genes, *mo-dnaK* and *mo-uvrA*, have completely different ORFs. These two genes showed a high degree of similarity to the reference genes (90 and 81%, respectively), indicating that these genes were found in the metagenomic library. The G + C content of the inserts was 49.9% and 51.7%, respectively. According to the NCBI BLAST results, *mo-uvrA* belonged to the *Firmicutes* phylum, *S. gallolyticus subsp*. This is a gram-negative facultative anaerobic bacterium that encodes an ATP-dependent DNA repair enzyme, which results in a bacteria that is resistant to acid. By contract, *Mo-dnaK* encodes HSP70 protein, which belongs to the family and heat shock proteins, involved in various biological activities.

**Table 2:** Bioinformatics analysis of the mo-uvrA and mo-dnaK

Code	mo-dnaK	mo-uvrA
Seq length (bp)	1956	2826
Site^a^(Nt range^b^)	1–83aa (835–398 bp)	1–743aa (382–2936 bp)
G + C %	48.7%	46.9%
Alignment^c^	*Streptococcus suis*	S*. pasteurianus* ATCC 43,144
Protein(accession No.)	HSPs70 (EGF09631)	ABC transporter protein (WG_0056)
E-Value	0	0
Identities %	438/539 (81%)	2622/2826 (90%)
Putative function^d^	Trigger protease-oriented pathways for the elimination	Maintain the pH inside the cell
Throughout	of the damaged polypeptides	The pumping function
Possible transmembrane helices^e^	0	0

^a^Protein range (site) of alignment into the known proteins

^b^Nucleotide range (nt range) of the predicted ORF within insert

^c^Most similar protein, currently identified with blast

^d^Most similar protein: putative function

^e^Results from the online server

*bp* base pair, *aa* amino acids, *score* bit score of the alignment using BLAST

### Acid resistance test results

Acid resistance was quantified using clones of the inserts. All groups of competent E. coli (*mo-uvrA* and *mo-dnaK* cloned groups, negative control group, and positive control group) were tested (Fig. 2c–e). All of the differences in acid resistance between the groups were statistically significant, except for that of the *mo-uvrA* cloned group versus the *mo-dnaK* cloned group. The results indicated that the two cloned and inserted genes significantly enhanced the acid resistance of the host; however, the degree of improvement between them was significantly different. Moreover, *mo-uvrA* exhibited the highest acid resistance (P < 0.001). Not only was the acid resistance imparted further verified, but also *mo-uvrA* underpinned the exploration of other heterologous acid-tolerant genes in E. coli (Fig. 2c–e).

## Discussion

Probiotics account for a large proportion of human oral microbiota: they are able to inhibit oral pathogenic microbiota, assist in the food metabolism, and regulate the immune system (Selwitz et al. 2007). Therefore, they are widely involved in the daily activities of the human body. In the past, scholars have often relied on the use of antibiotics for the treatment of caries. However, in addition to inhibiting the pathogenic microorganisms of caries, antibiotics also affect oral probiotics. Therefore, the ability to accurately eliminate the pathogenic microbiotas of caries without decreasing the levels of oral probiotics is one of the main goals of scholars in this field. The oral micro-environment of caries is often acidic (Dodds et al. 1986). This is beneficial to the survival of acid-resistant caries pathogens, wherein oral probiotics that are not acid-resistant will be suppressed. The development of precision medicine to target microbial acid resistance genes, thereby stopping caries development, while also effectively protecting oral probiotics, would provide a solution to caries without any detriment on health. Previous studies on the molecular and physiological responses of microbiotas to acid stimulation have been limited to cultured oral bacteria, such as *Streptococcus mutans* and *Helicobacter pylori* (Selwitz et al. 2007). However, the same effect on non-cultivable oral microbiota has not yet been extensively studied. As a vital technology for exploring the genetic and functional information of microbiota (including unknown genes and unknown microbiota), metagenomics technology is highly valuable to oral microbial research.

**Fig. 4:**
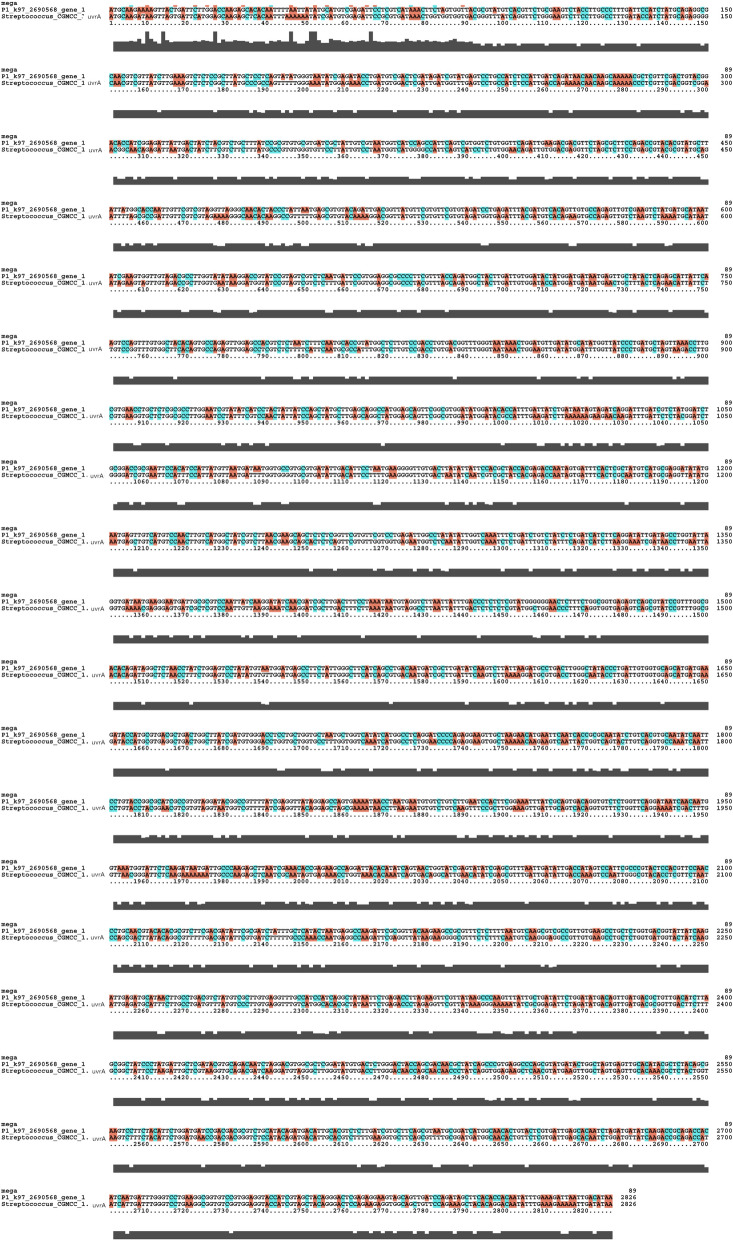
Structural differences between uvrA and mo-uvrA sequences

**Fig. 5:**
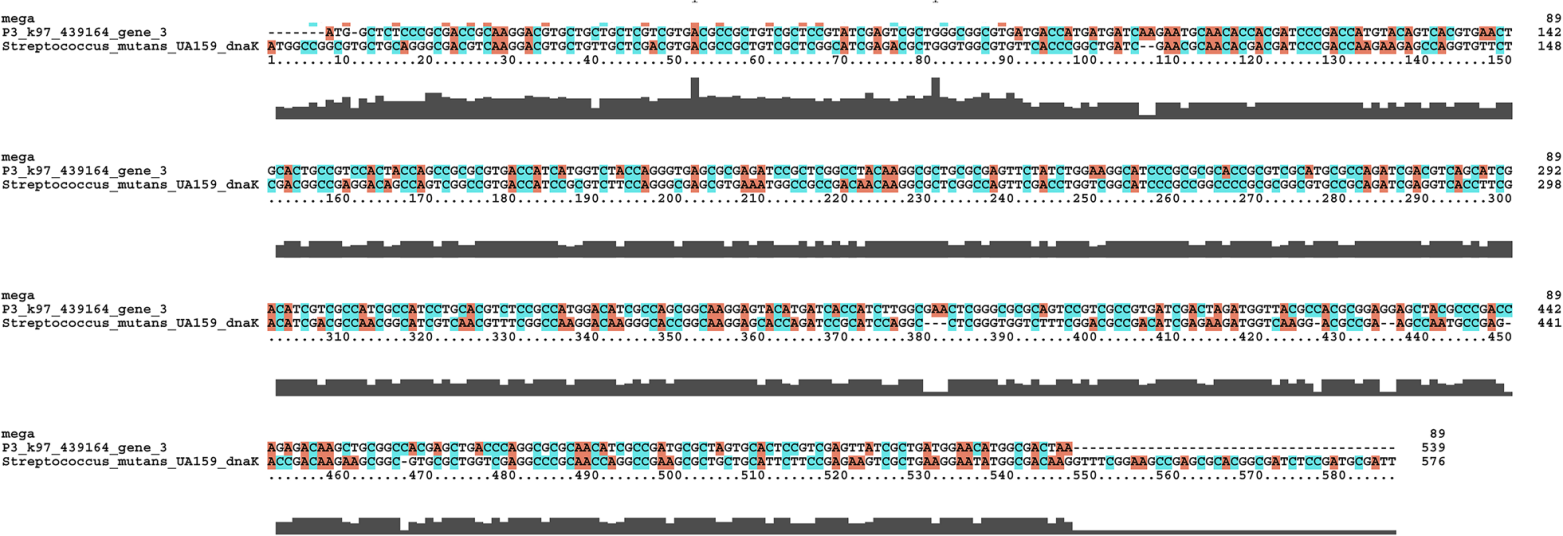
Structural differences between dnaK and mo-dnaK sequences

In the existing literature, the *uvrA* gene has been reported to act as an inducer of the production of AP endonuclease to enhance the acid resistance of *S. mutans* (Fig. 1a) (Jiang et al. 2009). Acid-resistant *uvrA* triggers *S. mutans* to rescue minor DNA damage resulting from acid etching through base excision repair. In addition, some scholars have identified *urvA* gene expression in *S. mutans* using differential display PCR when cells are grown at pH 7 and pH 5. In the absence of *uvrA*, the survival rate of *S. mutans* was found to be down-regulated. On the other hand, other groups have found that the *S. mutant* gene *danK* (Fig. 1a) contributes to the production of F-F0-ATPase (Morita et al. 2008), thus helping cells maintain a functional intracellular pH by removing protons from the cytoplasm. Interestingly, studies on E. coli have demonstrated that *dnaK* can enhance the stability of *uvrA* (Jayaraman et al. 1997). Due to the significant roles of these two genes, they were adopted in our study. According to our BLAST search results, 35 unknown gene sequences similar to those of *dnaK* were screened (Fig. 1c). For the *uvrA* gene, the corresponding local BLAST results indicated 19 unknown gene sequences with structures identical to that of the original gene (Fig. 1c). As with the principle of the start codon ATG and the stop codons TAA, TAG, and TGA, P3_k97_439164_gene_3 (or mo-*dnaK*) was selected, with a 81% similarity to the original *dnaK* gene, for subsequent experiments (Fig. 4). Similarly, P1_k97_2690568_gene_1 (or mo-*urvA*) was selected, with a 90% similarity to the original *urvA*, for further testing (Fig. 5). After cloning the two genes from the samples, they were transformed into competent *E. coli* for acid-resistance testing. As a result, the two cloned genes similar to *dnaK* and *urvA* were found to markedly render the bacteria resistant to acids (Fig. 2c–e).

According to the acquired gene sequences, the *mo-uvrA* gene obtained in our study was aligned with sequences from the *Firmicutes* phylum, *S. gallolyticus* subsp., and S*. pasteurianus* ATCC 43,144 non-cultivable oral microbiota (Table 2). Bioinformatics analysis revealed that *Mo-uvrA* contains two domains: the *Mo-uvrA* interaction domain and the *uvrA* DNA-binding domain (encoded by ORF8), which encodes an ATP-dependent DNA repair enzyme (Table 2). The complete gene sequence is essential for maintaining the function of bacteria (Zou et al. 1998). When gene sequences from the microbiota are damaged in an acidic environment, the ATP-dependent DNA repair enzyme quickly cuts the intact base pair and repairs the damaged DNA through the BER pathway. At the same time, it recruits ATP ligase to the site of DNA damage and attracts short pieces of DNA to fill the damage site before repair (Gross et al. 2012). In our experiments, *mo-uvrA* was transformed into competent *E. coli* in a harsh acidic environment (pH 1.9) and was found to exhibit strong acid resistance compared with the control group (Fig. 2c–e). However, whether the ATP-dependent DNA repair enzyme plays a key role requires further study. On the other hand, we also found that this gene encodes the ABC transporter protein, which allows for ATP binding to maintain the pH inside the cell throughout the pumping function (Martin et al. 2002). All of the aforementioned mechanisms make microbiota acid-resistant (Table 2).

Another positive clone, *mo-dnaK*, which encodes the HSP70 protein, is involved in multiple organismal activities. Previous studies have shown that the HSP family is a group of highly conserved proteins that exist widely in bacteria, accounting for 5 to 10% of the total proteins in a bacterial host. Once the bacteria are stimulated by certain factors, such as a rise in temperature, an oxidation reaction, and chemical or acid stimulation, HSP expression will exceed 15% (Weller et al. 2001). In response to these stimuli, HSPs first trap the partially folded client protein to avoid aggregation and then deliver it to ATP-dependent HSPs (such as HSP 70), either to refold the client protein and send it to proper cellular locations or to trigger protease-oriented pathways for the elimination of the damaged polypeptides. HSPs can immediately exert protective effects by retaining the vitality of bacteria and repairing the damage (Tanabe et al. 2006). A previous study showed that the downregulation of HSP70 severely damaged the colon mucosa of mice after acid attack (Schlesinger et al. 1990). Similar responses were observed in the acidic environment of the intestines of patients with inflammatory bowel disease (IBD), where downregulated HSP70 exacerbated the clinical severity of this disease (Lindquist et al.^1988^; Liu et al. 2014). Some studies have also identified several interactions between HSP70 and microbiotas. For example, studies on *Lactobacillus rhamnosus* GG and VSL3 have found that the up-regulation of HSP70 can significantly increase the barrier function of the cell wall, reduce permeability, and maintain the stability of the pH value in cells. Studies on *Lactobacillus* have also shown that HSP70 expression regulates proton flow inside and outside cells and ensures a stable acidic environment (Liedel et al. 2011; Tanaka et al. 2009). This indicates that the acid-resistant effect of over expressed HSP70 may contribute to protecting oral microbiota against dental caries. However, further research is still needed to confirm these findings (Table 2).

In our study, a small number of functional genes were identified and analyzed using metagenomics. Since the establishment of a metagene library using BLAST to pinpoint unknown acid-resistant genes is a novel method, our experiments contain several limitations that are worth mentioning. Firstly, some genes are subunits encoding a multiunit enzyme and cannot make competent *E. coli* acid-resistant after cloning. Secondly, in competent *E. coli* cells, the transfer and expression of cloned genes may fail due to the fact that some Gram-positive bacterial proteins are toxic in hosts. Thirdly, limitations exist in our sampling and analytical methods: (1) only the genes of *S. mutans* were used; (2) the sample size was small, with saliva samples obtained from thirty patients; (3) oral microbial samples of patients with dental caries were used – the small sample size limits the integration of genetic information into the metagenomic library, which reduces its accessibility to more functional genes; (4) operationally, our experiments are accompanied by a certain degree of uncontrollability, particularly in the process of gene cloning and vectors, which may result in the partial or complete loss of important biological information (Additional files 1, 2, 3).

In conclusion,we identified unknown acid-tolerant genes *mo-dnaK* and *mo-uvrA* in the oral microbes of patients with dental caries using metagenomics. Our findings demonstrate the feasibility and efficiency of these experimental methods for functional gene research on oral microbiota. If subsequent experiments can confirm that all the unknown genes from blast have acid-resistance function, then these genes are good targets for caries precise treatment. The development of methods for the treatment of dental caries while preventing the loss of oral probiotics is deserving of further study,the metagene library established in our study provides a resource for the future scholars (Additional files 4, 5).

## Supplementary Information

**Additional file 1.** Streptococcus mutans UA159 DnaK (dnaK) gene; Amino acids.

**Additional file 2.** Streptococcus salivarius strain uvrA gene, complete cds; Amino acids.

**Additional file 3.** P3_k97_439164_gene_3, with a length of 1956 base pairs, a score of 418 bits (226), and 81% similarity to the original danK gene.

**Additional file 4.** P1_k97_2690568_gene_1, with a length of 2826 base pair, a score of 4089 bits (2214), and 90% similarity to the original uvrA.

**Additional file 5.** A total of 35 unknown gene sequences with structures similar to those of dnaK were found; 13 unknown gene sequences with structures similar to the original uvrA were found using local BLAST analysis. **Table.a** Metagenomic sequencing data. **Table.b** Bioinformatics Analysis of the mo-uvrA and mo-dnaK.

## Data Availability

Sequence data associated with this project have been deposited in the NCBI (https://www.ncbi.nlm.nih.gov/) Short Read Archive database (NCBI accession no. PRJNA533520).

## References

[CR1] Agarwal V, Blanton JM, Podell S, Taton A, Schorn MA (2017). Metagenomic discovery of polybrominated diphenyl ether biosynthesis by marine sponges. Nat Chem Biol.

[CR2] Dodds MW, Edgar WM (1986). Effects of dietary sucrose levels on pH fall and acid-anion profile in human dental plaque after a starch mouth-rinse. Archoral Biol.

[CR3] Gilbert JA, Dupont CL (2010). Microbial metagenomics: beyond the genome. Annu Rev Mar Sci.

[CR4] Gross EL, Beall CJ, Kutsch SR, Firestone ND, Leys EJ (2012). Beyond Streptococcus mutans: dental caries onset linked to multiple species by 16S rRNA community analysis. PLoS ONE.

[CR5] Jain KK (2002). Personalized medicine. Curr Opin Mol Ther.

[CR6] Jayaraman GC, Penders JE, Burne RA (1997). Transcriptional analysis of the Streptococcus mutans hrcA, grpE and dnaK genes and regulation of expression in response to heat shock and environmental acidification. Mol Microbiol.

[CR7] Jiang Y, Rabbi M, Kim M, Ke C, Lee W, Clark RL (2009). UVA generates pyrimidine dimers in DNA directly. Biophys J.

[CR8] Jin J, Liu S, Zhao L, Ge K, Mao X, Ren F (2011). Changes in ffh, uvrA, groES and dnaK mRNA abundance as a function of acid-adaptation and growth phase in Bifidobacterium longum BBMN68 isolated from healthy centenarians. Curr Microbiol.

[CR9] Koponen J, Laakso K, Koskenniemi K, Kankainen M, Savijoki K (2012). Effect of acid stress on protein expression and phosphorylation in Lactobacillus rhamnosus GG. J Proteomics.

[CR10] Liedel JL, Guo Y, Yu Y, Shiou SR, Chen S (2011). Mother's milk-induced Hsp70 expression preserves intestinal epithelial barrier function in an immature rat pup model. Pediatr Res.

[CR11] Lindquist S, Craig EA (1988). The heat-shock proteins. Annu Rev Genet.

[CR12] Liu H, Dicksved J, Lundh T, Lindberg JE (2014). Heat shock proteins: intestinal gatekeepers that are influenced by dietary components and the gut microbiota. Pathogens.

[CR13] Loesche WJ (1986). Role of Streptococcus mutans in human dental decay. Microbiol Res.

[CR14] Martin IV, MacNeill SA (2002). ATP-dependent DNA ligases. Genome Biol.

[CR15] Morita R, Nakagawa N, Kuramitsu S, Masui R (2008). An O 6-methylguanine-DNA methyltransferase-like protein from Thermus thermophilus interacts with anucleotide excision repair protein. J Biochem.

[CR16] Peterson SN, Meissner T, Su AI, Snesrud E, Ong AC, Schork NJ, Bretz WA (2014). Functional expression of dental plaque microbiota. Front Cell Infect MI.

[CR17] Reshkin SJ, Greco MR, Cardone RA (2014). Role of pH, and proton transporters in oncogene-driven neoplastic transformation. Philos Trans R Soc B.

[CR18] Rosier BT, Marsh PD, Mira A (2018). Resilience of the oral microbiota in health: mechanisms that prevent dysbiosis. J Dent Res.

[CR19] Schlesinger MJ (1990). Heat shock proteins. J Biol Chem.

[CR20] Selwitz RH, Ismail AI, Pitts NB (2007). Dental caries. Lancet.

[CR21] Song C, Wang B, Tan J, Zhu L, Lou D (2017). Discovery of tauroursodeoxycholic acid biotransformation enzymes from the gut microbiome of black bears using metagenomics. Sci Rep UK.

[CR22] Tanabe M, Atkins HS, Harland DN, Elvin SJ, Stagg AJ, Brown KA (2006). The ABC transporter protein OppA provides protection against experimental Yersinia pestis infection. Infect Immun.

[CR23] Tanaka KI, Mizushima T (2009). Protective role of HSF1 and HSP70 against gastrointestinal diseases. Int J Hyperther.

[CR24] Wade WG (2013). The oral microbiome in health and disease. Pharmacol Res.

[CR25] Weller GR, Doherty AJ (2001). A family of DNA repair ligases in bacteria?. FEBS Lett.

[CR26] Zhang M, Zheng Y, Li Y, Jiang H, Huang Y, Du M (2018). Acid-resistant genes of oral plaque microbiome from the functional metagenomics. J Oral Microbiol.

[CR27] Zou Y, Crowley DJ, Van Houten B (1998). Involvement of molecular chaperonins in nucleotide excision repair DnaK leads to increased thermal stability of UvrA, catalytic UvrB loading, enhanced repair, and increased UV resistance. J Biol Chem.

