# Supplementary figures and images for: Identification of unknown acid-resistant genes of oral microbiotas in patients with dental caries using metagenomics analysis
Source: AMB Express. 2021 Mar 6;11:39. doi: 10.1186/s13568-021-01199-4 (PMC7936999; doi:10.1186/s13568-021-01199-4)

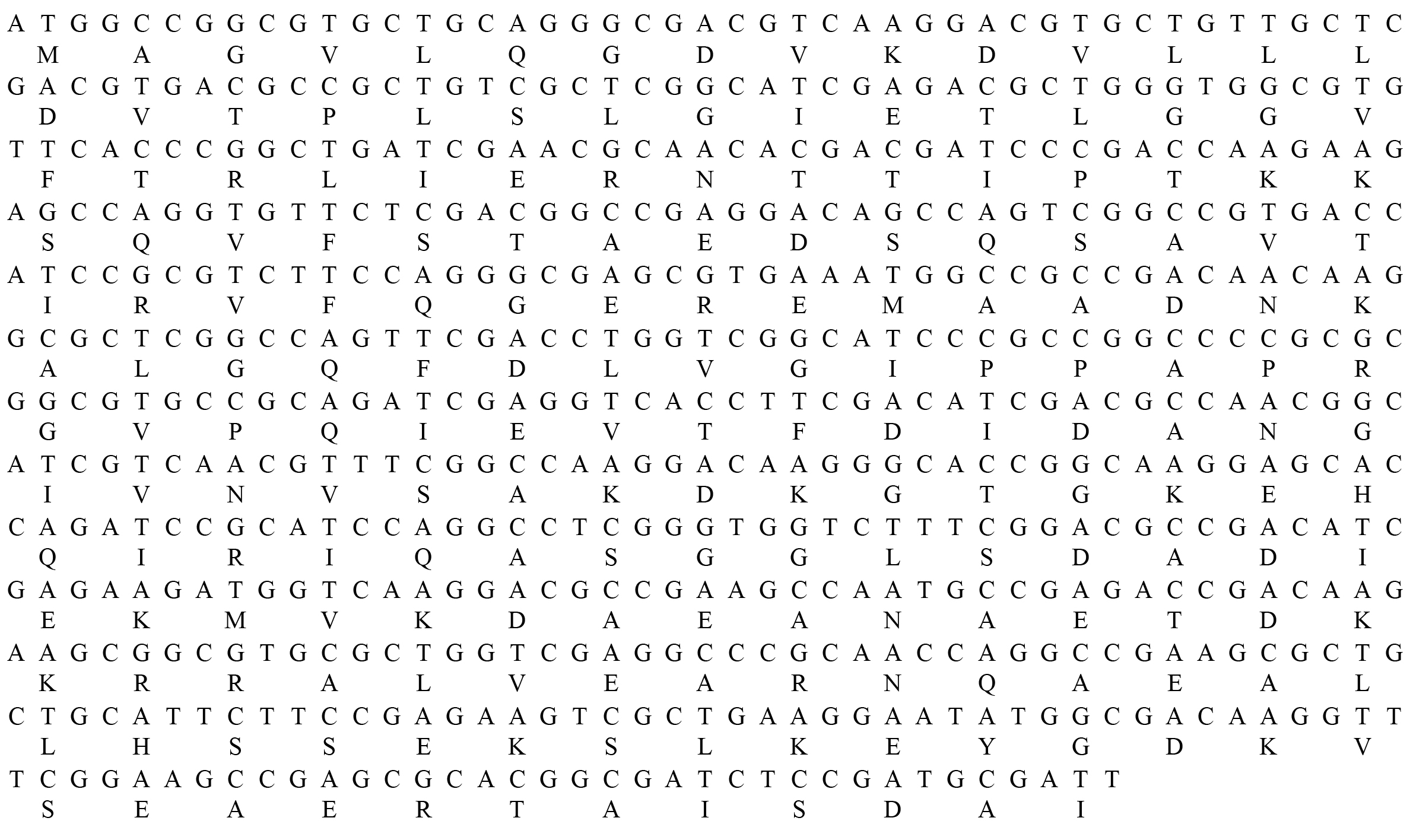

Supplement: Supplementary file 1 — Additional file 1. Streptococcus mutans UA159 DnaK (dnaK) gene; Amino acids. [file 13568_2021_1199_MOESM1_ESM.tif]

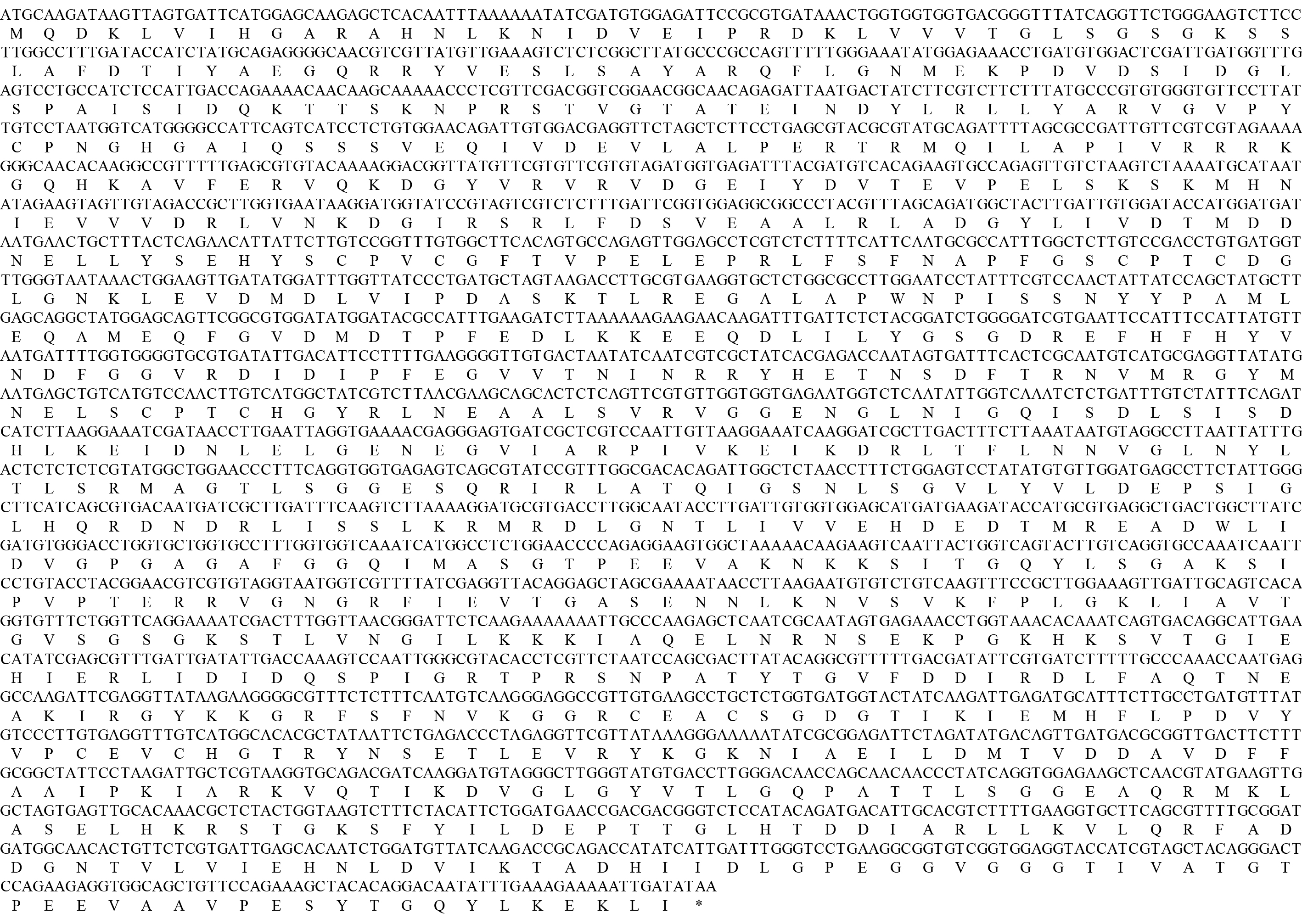

Supplement: Supplementary file 2 — Additional file 2. Streptococcus salivarius strain uvrA gene, complete cds; Amino acids. [file 13568_2021_1199_MOESM2_ESM.tif]

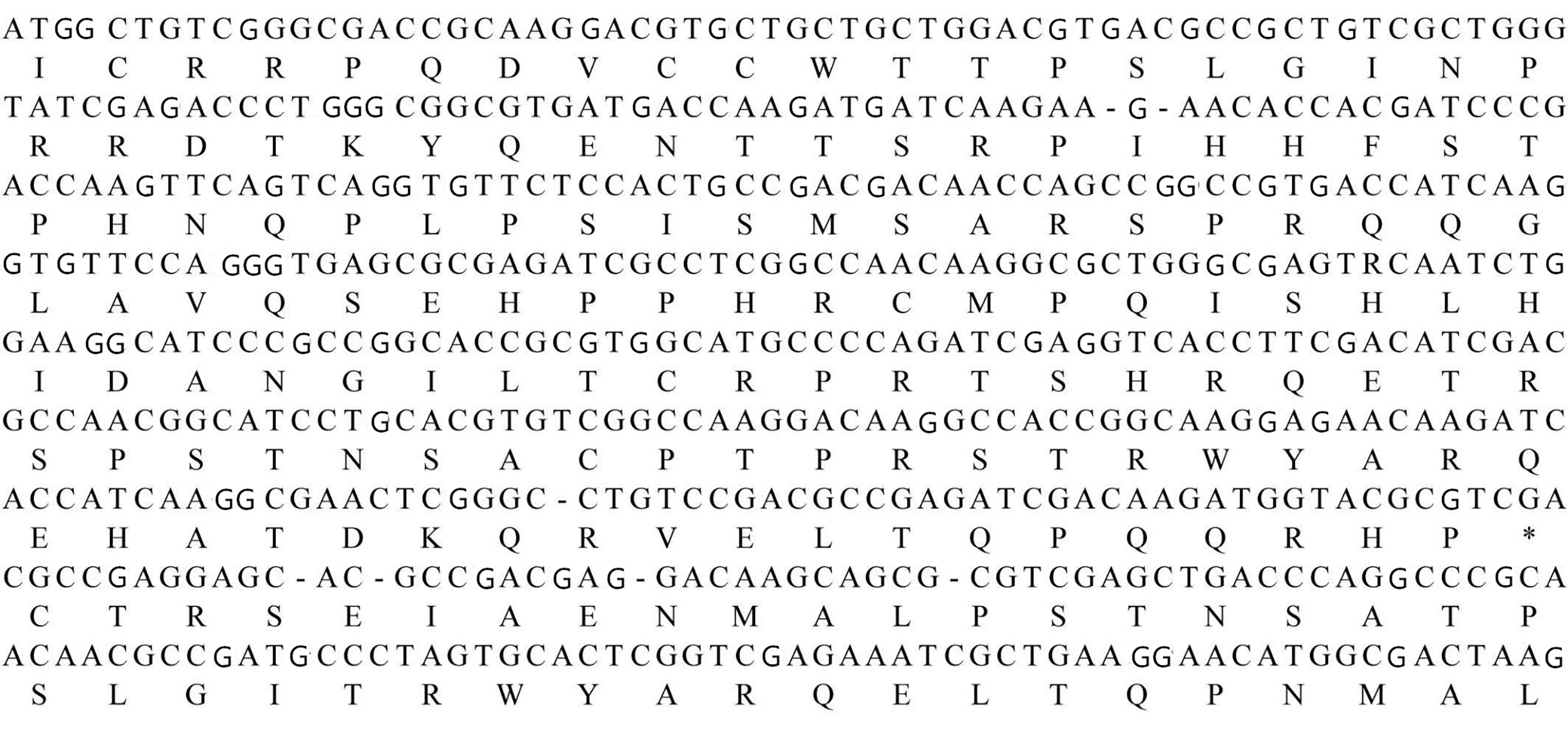

Supplement: Supplementary file 3 — Additional file 3. P3_k97_439164_gene_3, with a length of 1956 base pairs, a score of 418 bits (226), and 81% similarity to the original danK gene. [file 13568_2021_1199_MOESM3_ESM.tif]

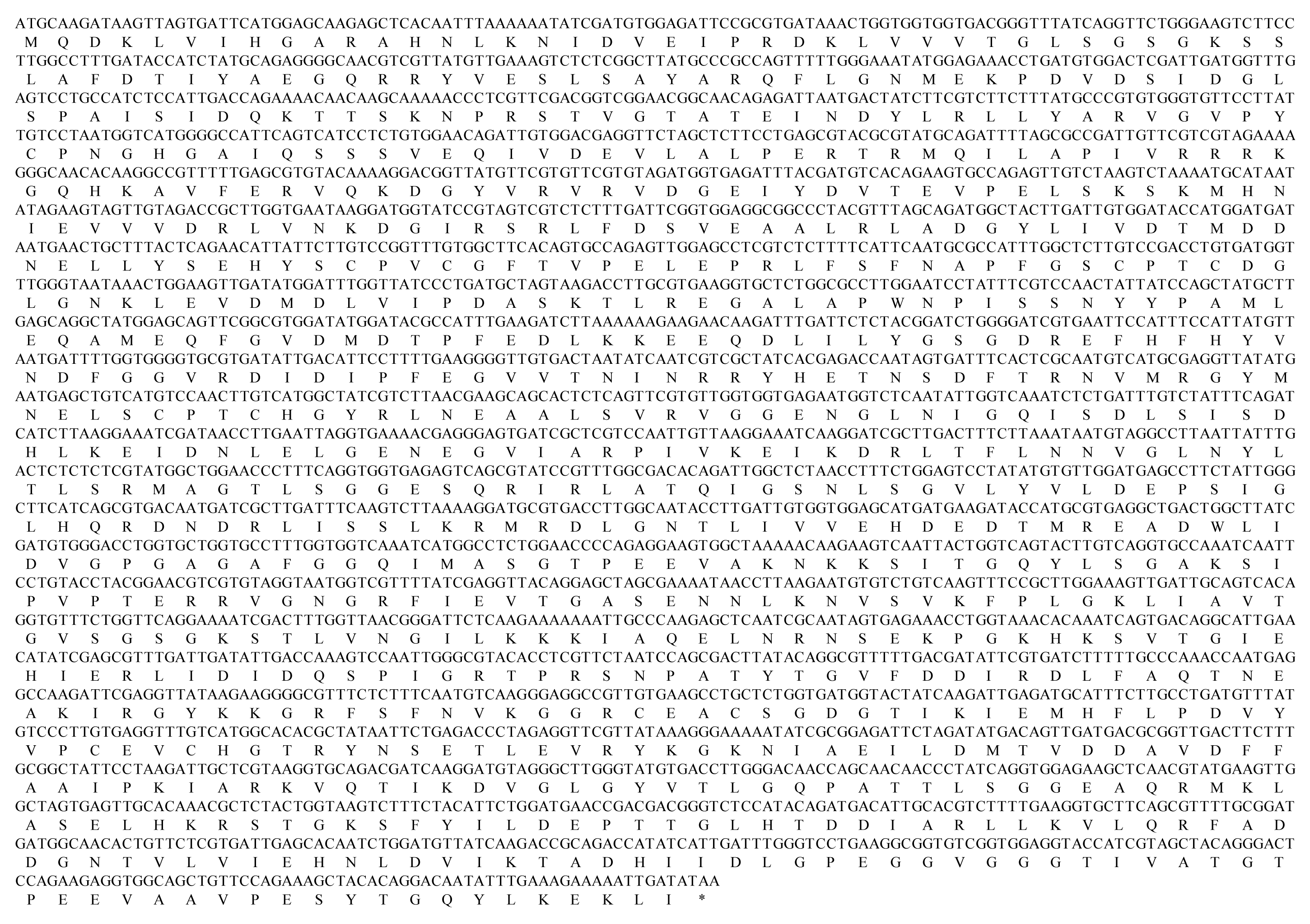

Supplement: Supplementary file 4 — Additional file 4. P1_k97_2690568_gene_1, with a length of 2826 base pair, a score of 4089 bits (2214), and 90% similarity to the original uvrA. [file 13568_2021_1199_MOESM4_ESM.tif]

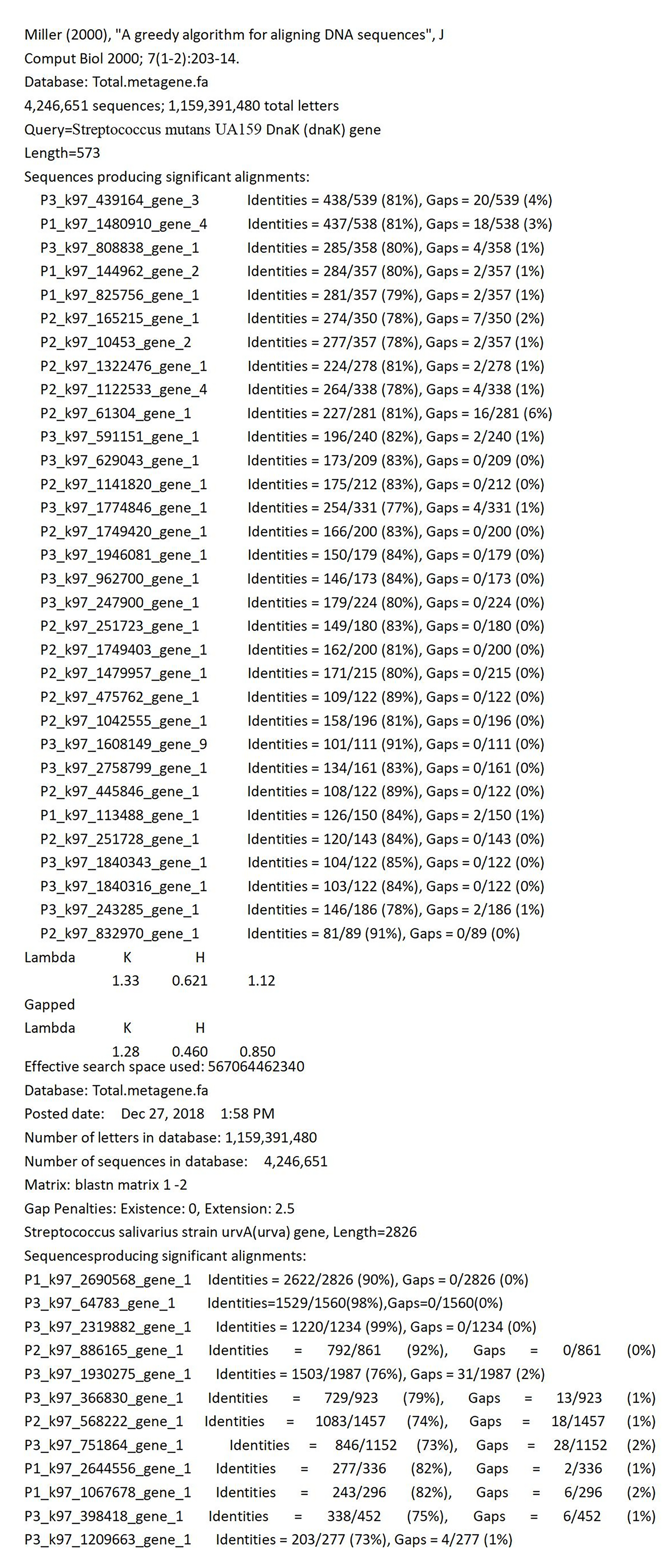

Supplement: Supplementary file 5 — Additional file 5. A total of 35 unknown gene sequences with structures similar to those of dnaK were found; 13 unknown gene sequences with structures similar to the original uvrA were found using local BLAST analysis. Table.a Metagenomic sequencing data. Table.b Bioinformatics Analysis of the mo-uvrA and mo-dnaK. [file 13568_2021_1199_MOESM5_ESM.tif]
